# *Schizosaccharomyces pombe* Assays to Study Mitotic Recombination Outcomes

**DOI:** 10.3390/genes11010079

**Published:** 2020-01-10

**Authors:** Hannah M. Hylton, Bailey E. Lucas, Ruben C. Petreaca

**Affiliations:** 1 Biology Program, The Ohio State University, Marion, OH 43302, USA; hylton.167@buckeyemail.osu.edu; 2 The Ohio State University Comprehensive Cancer Center, Columbus, OH 43210, USA; lucas.637@osu.edu; 3 Department of Molecular Genetics, The Ohio State University, Marion, OH 43302, USA

**Keywords:** mitotic recombination, DNA replication, double strand break (DSB), chromosomal rearrangements

## Abstract

The fission yeast—*Schizosaccharomyces pombe*—has emerged as a powerful tractable system for studying DNA damage repair. Over the last few decades, several powerful in vivo genetic assays have been developed to study outcomes of mitotic recombination, the major repair mechanism of DNA double strand breaks and stalled or collapsed DNA replication forks. These assays have significantly increased our understanding of the molecular mechanisms underlying the DNA damage response pathways. Here, we review the assays that have been developed in fission yeast to study mitotic recombination.

## 1. Introduction

An accurate DNA damage response (DDR) is fundamentally important for cellular homeostasis. The DDR involves first detection of the damage and activation of the DNA damage checkpoints, then recruitment of the repair machinery and repair of the damage. In humans, defects in DDR can lead to a variety of syndromes and diseases [1].

The DNA double strand break (DSB) constitutes severing of the chromosome into two parts. Threatening the viability of a cell, DSBs can lead to loss of essential chromosomal regions. In multicellular organisms, unrepaired breaks may cause cells to undergo apoptosis and new cells can be regenerated [2,3]. However, inappropriate repair of breaks may give rise to translocations, deletions, duplications and inversions [4,5] which have been identified in cancer cells [6].

DSBs may be produced by endogenous or exogeneous sources but are generally repaired by the same mechanisms [7]. Most endogenous breaks occur as a consequence of replication stress. DNA replication forks can stall or collapse as they pass through heterochromatin regions [8,9], collide with RNA polymerases [10] or pass through other fragile sites characterized by repetitive elements or euchromatin to chromatin boundaries [11,12]. The recombination machinery evolved to rescue stalled or collapsed replication forks [13]. 

**Figure 1:**
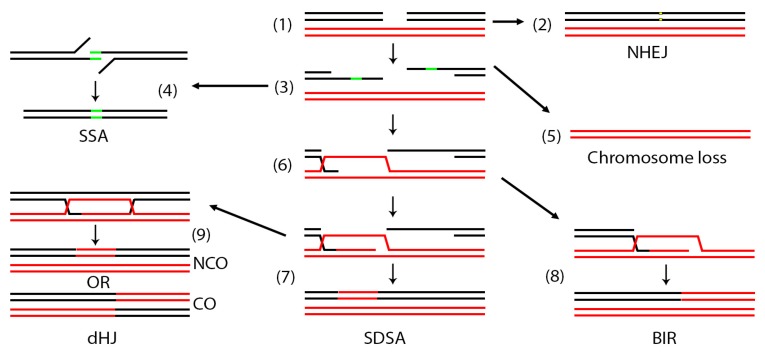
Cellular pathways of mitotic DNA double strand break repair. In a diploid cell, a DSB may occur in one of the two homologous chromosomes (1). The DSB may be repaired by non-homologous end joining (NHEJ) (2). When repair occurs by homologous recombination (HR), the DSB is first resected (3) to expose areas of single stranded DNA. If direct repeats (green areas) exist on the same chromosome, the break may be repaired by single strand annealing (SSA) (4). If homology is not found, the chromosome may be entirely lost (5). When homology is found elsewhere, the broken ends may invade this region (6). In synthesis dependent strand annealing (SDSA) (7) the invading strand may copy a small region then release and re-anneal. In break induced replication (BIR) (8) the invading strand may copy to the end of the red chromosome. In this case the right part of the broken black chromosome is lost. Occasionally, a more complex double Holiday junction (dHJ) may be established (9), the resolution of which can result in crossovers (CO) or non-crossovers (NCO).

The multitude of repair mechanisms that eukaryotic cells evolved to deal with these breaks have been previously extensively reviewed [7,10,14,15,16,17,18,19] and can be generally broken into two major pathways, non-homologous end joining (NHEJ) and homologous recombination (HR). NHEJ involves localized repair of breaks with no major sequence rearrangements. Repair can result in small deletions or alterations of the sequences neighboring the break so NHEJ is considered an error-prone form of repair [20]. HR can be subdivided into several mechanisms that are both genetically and biochemically related such as single strand annealing (SSA), break induced replication (BIR) and the two closely related gene conversion (GC) mechanisms: synthesis dependent strand annealing (SDSA) and double Holliday junction (dHJ) (Figure 1) [7]. HR has been traditionally considered error proof because it uses an intact template sequence to copy the missing or broken region although some pathways of HR can be quite mutagenic [21].

*Schizosaccharomyces pombe* (fission yeast) diverged from *Saccharomyces cerevisiae* (budding yeast) approximately a billion years ago [22,23]. Many genes have been identified in *S. pombe* that show similarity to genes involved in human disease [24]. This is comparable to the level of conservation between *S. cerevisiae* and humans [25]. However, *S. pombe* appears to show higher conservations in chromosome structure and function genes [26] making *S. pombe* a great model system for studying chromosomal dynamics.

Specific to the study of chromosomal double strand break repair, fission yeast is great for several reasons. First, DDR genes are highly conserved from yeast to humans [27,28]. Second, in fission yeast, repetitive DNA elements, which are often a reason for endogenous breaks in human cells [29], are found only at centromeres and telomeres [30]. The overall structure of centromeres is conserved between yeast and humans. We and others have previously shown that replication trough the centromere does lead to chromosome breakage [31,32,33,34,35]. The presence of these repeats in only a few well characterized regions allows the better monitoring of the events that govern DSB repair. Third, haploid fission yeast has only three chromosomes [36] making it easier to track translocations and other aberrations resulting from inappropriate break repair. Fourth, fission yeast is as well suited for genetic screens as baker’s yeast [37]. Finally, working with fission yeast is inexpensive and easy to learn.

A plethora of assays have been developed to study DNA damage in various systems and some are described in several recent elegant reviews [38,39,40,41]. Here, we focus on *S. pombe* and review primarily the in vivo assays that have been developed in this system to study mitotic recombination. We briefly summarize these assays and direct the reader to the original publications for more details.

## 2. Mini-Chromosome Assays

**Figure 2:**
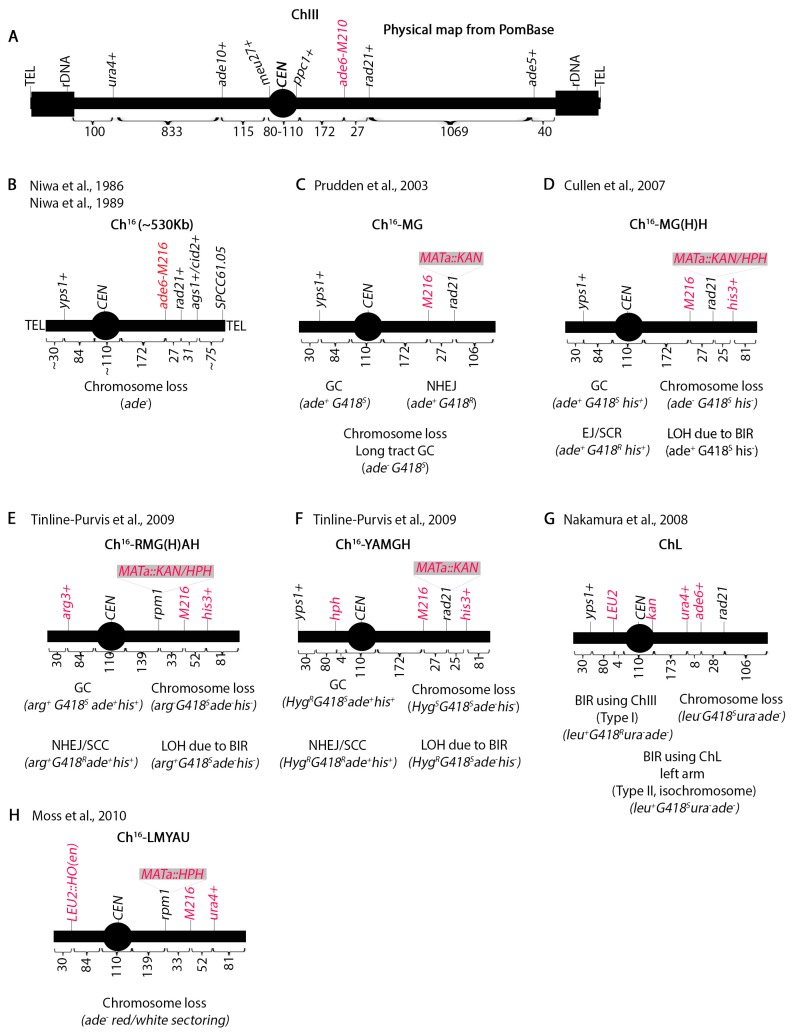
*S. pombe* mini-chromosome derivatives of Ch.III to study recombination. (**A)** Diagram of Chromosome III indicating the physical position of some genes. The numbers indicate kilobases. *ade6-M210* provides intra-allelic complementation with *ade6-M216*. Ch.III is approximately 3.5 Mb. **(B**) The Ch^16^ mini-chromosome isolated by Niwa et al. Ch^16^ is 0.5 Mb and lacks rDNA repeats. Chromosome loss can be monitored by appearance of adenine auxotrophs. (**C**) Ch^16^-MG designed by Prudden et al. with the HO endonuclease restriction site at the *rad21* locus. This chromosome can monitor, gene conversion, NHEJ and chromosome loss. (**D**) Modified Ch^16^-MG to include the *his3^+^* marker close to the right telomere which can monitor LOH due to BIR. (**E**) The *arg3+* marker was placed on the left arm of Ch^16^-MGH to produce Ch^16^-RMGAH. The *arg3+* marker allows monitoring of extensive BIR that leads to loss of all markers on the right arm but retention of the left arm. (**F**) This mini-chromosome was generated to show that the outcomes of the assay in **E** were not dependent on break position. (**G**) A mini-chromosome designed to investigate recombination at centromeric repeats. (**H**) A mini-chromosome to investigate chromosome loss by red/white sectoring. The HO endonuclease is integrated in the left arm of the chromosome and marked with *LEU2*.

Mini-chromosome assays have been instrumental in elucidating many recombination pathways. Chromosome III (Ch.III), the smallest *S. pombe* chromosome, houses rDNA repeats at both ends next to the telomeres and has the longest centromere [36] (Figure 2A). Using γ irradiation, Niwa et al. [42,43] isolated a truncated Ch.III (termed Ch^16^) in haploid yeast (Figure 2B). Unlike *S. cerevisiae* which has point centromeres [44], *S. pombe* centromeres are characterized by repetitive elements resembling higher metazoans [30]. Since both the centromere and the rDNA repeats fluctuate in number between strains, the size of Ch.III cannot be determined precisely making it approximately 3.5 Mb. Initial genetic maps estimated the centromere to be 100 Kb [45,46,47] but latter structural analysis identified a 110 Kb centromere [48]. The fission yeast genome sequence [24] set the centromere size at 68 Kb with the caveat that seven 6760 bp repeats were missing. The addition of these repeats to the 68 Kb region brings the size of CEN III to 110 Kb which is what PomBase (the scientific database for fission yeast) reports [49,50]. The centromere spans between the *meu27^+^* and *ppc1^+^* loci (Figure 2A).

The truncated mini-chromosome Ch^16^ should retain an intact Ch.III centromere but not have rDNA repeats. Pulse field gel electrophoresis shows that this mini-chromosome is about 530 Kb [42,43,51]. The chromosome can be maintained in haploid yeast by intra-allelic complementation between *ade6-M210* (Ch.III) and *ade6-M216* (Ch^16^) (Figure 2A,B). Genetic mapping by Niwa et al. [42] showed that the left arm does not include the *ade10^+^* locus but the *yps1^+^* locus is present [51]. There are approximately 30 Kb between *yps1^+^* and *ade10^+^* and 84 Kb between *yps1^+^* and *meu27^+^*, so it appears that the left arm is about 110 Kb. On the right arm, *ade6^+^* is 172 Kb from *ppc1^+^*. Genetic mapping shows the presence of the *ags1^+^* locus (formerly *tps16^+^* [52]) 58 Kb telomere proximal from *ade6^+^* but not *tps14^+^* which is approximately 200 Kb from *ags1^+^* [42]. *cid2^+^* is within 5 Kb of *ags1^+^*. Southern blotting has shown the presence of the SPCC61.05 locus 75 Kb from *ags1^+^* [53]. Thus, the right arm is about 305 Kb.

Niwa et al. determined that Ch^16^ is stable in mitotically dividing haploid cells at one copy per cell but unstable at two copies [42]. Remarkably, cells were able to simultaneously stably propagate both Ch^16^ and a 100 Kb shorter derivative (Ch^16D1^) suggesting that yeast cells may be able to determine ploidy by the size of the chromosome. Ch^16^ loss monitored by appearance of adenine auxotrophs occurred at a frequency of 1 in 10^4^ cells.

The Humphrey lab has produced several derivatives of Ch^16^ to study recombination outcomes in vivo. They initially placed the *S. cerevisiae* homothallic endonuclease (*HO*) restriction site (*MATa*) marked with the kanamycin antibiotic resistance gene (*KAN*) at the *rad21^+^* locus 27 Kb telomere distal of *ade6-M216* producing Ch^16^-MG (Figure 2C) [54]. *KAN* confers resistance to G418 (Geneticin). Upon induction of a single DSB, gene conversion of the *KAN* locus can be monitored by screening for adenine prototrophic and G418 sensitive (ade+G418^S^) colonies while chromosome loss will lose both markers. Long track gene conversion produces the same phenotype as chromosome loss, so it was necessary to distinguish between the two by pulse field gel electrophoresis (PFGE). Similarly, because an ade^+^G418^R^ phenotype could result from repair by NHEJ or failure of *HO* cutting, it was necessary to sequence across the DSB site.

Ch^16^-MG was improved by adding the *his3^+^* marker at the *cid2^+^* locus approximately 25 Kb from the *MATa* site creating Ch^16^-MGH (Figure 2D) [53]. The advantage of this is that BIR can be investigated by monitoring the loss of both *KAN* and *his^+^* markers but retention of the adenine prototrophy (ade+G418^S^his-). In BIR, the break is repaired by copying the missing information from Ch.III which is *his3^−^*. PFGE followed by southern blotting showed both a larger mini-chromosome termed *Ch^x^* and the presence of the *ade5*^+^ marker, which could have only been transferred onto the Ch^16^ from Ch.III by BIR. A mini-chromosome with *MATa-HPH* marker (Ch^16^-MHH) was also constructed which behaved identically to Ch^16^-MGH. HPH confers resistance to hygromycin abbreviated as HYG in this paper. Bioneer has generated a deletion mutant library marked with *KanMX* [55] and this Ch^16^-MHH chromosome is a powerful tool in screening genes that affect repair.

A third marker (*arg3^+^*) was introduced on the left arm of Ch^16^-MGH at the *yps1*^+^ locus 84 Kb from the *meu27^+^* locus. An additional modification was moving the *MATa* site 33 Kb left of the *ade6-M216* marker. This created Ch^16^-RMGAH (Figure 2E) [51] which can monitor extensive BIR that results in loss of all markers on the right arm but retention of the *arg3^+^* marker. Thus, the *arg3^+^* marker distinguishes.

Extensive BIR from chromosome loss. To ensure that the new placement of the *MATa* and *arg3^+^* marker on the left arm did not cause a locus-specific form of repair, they generated chromosome Ch^16^-YAMGH where the *MATa* site was left at the *rad21^+^* locus and an *HPH* marker was put 4 Kb from the centromere (Figure 2F). Remarkably, PFGE analysis of the arg+G418^S^ade-his- or HYG^R^G418^S^ade-his- colonies showed that the two outcomes were identical suggesting that the mechanism of repair was independent of the break position.

Further analysis showed that following break induction, telomere distal sequences of the break are lost while extensive resection of the centromere proximal sequences occurs. Inverted centromeric repeats facilitate formation of an isochromosome which results from invasion and duplication of both the centromere and the left arm of the chromosome onto the right [51]. Nakamura et al. [33] constructed the ChL mini-chromosome (Figure 2G) to study these isochromosomes and they found that they can occur in the absence of a break. Both groups showed that Rad51 suppresses isochromosome formation [35,51]. Using the ChL chromosome, we have also shown that perturbation of the replication machinery or the centromere heterochromatin increases these isochromosome events. This suggests that isochromosomes originate from improperly repaired random breaks that occur during replication [31]. Remarkably, we also identified some more drastic copy number variations in double chromatin and replication mutants which entirely lost Ch.III and acquired other smaller chromosome fragments. The structure and mechanism of these smaller chromosome fragments remains to be elucidated.

Finally, Ch^16^-LMYAU was generated to screen Bioneer mutants for chromosome loss phenotypes (Figure 2H) [56]. Previously, the *HO* endonuclease was propagated on a plasmid but here the investigators introduced it at the *yps1^+^* locus marked with the *S. cerevisiae LEU2*. The *ura4^+^* marker was placed at the *cid2^+^* locus. Chromosome loss was assayed by red/white sectoring in response to break induction. Detailed experimental procedures for using these assays were published [57]. Although we do not discuss it here, a chromosome loss assay in diploid cells has also been designed [58]. In addition to chromosome loss, this assay also monitors intra-homologue recombination.

## 3. Recombination at Repetitive Elements

**Figure 3:**
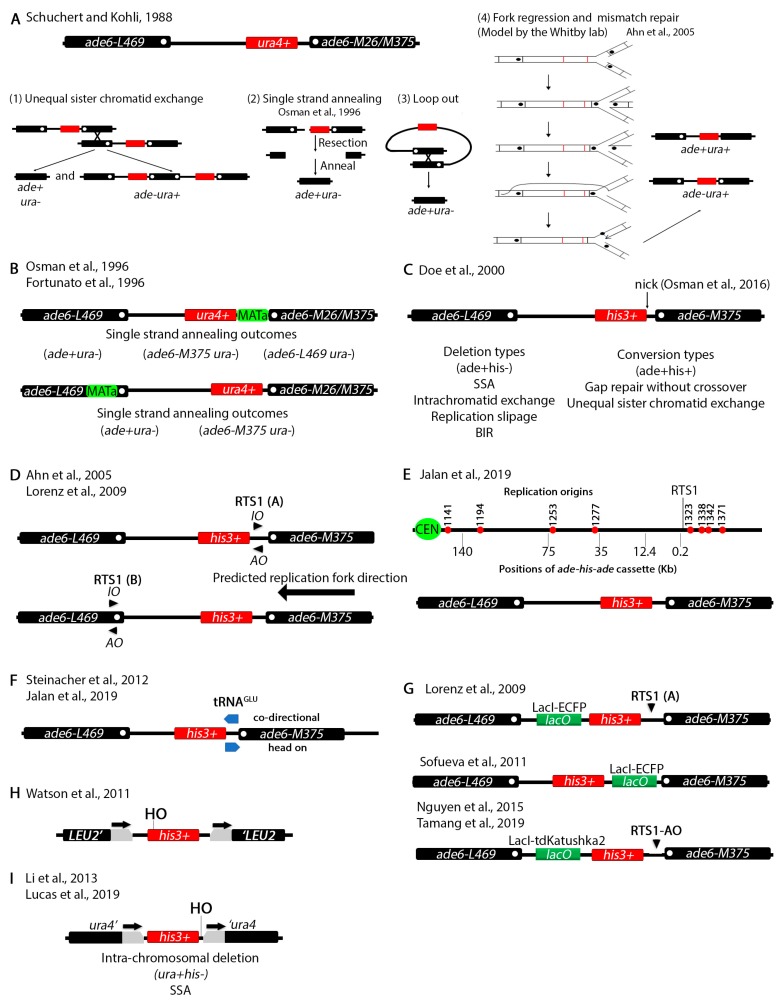
Recombination at tandem repeats. (**A**) An assay to study recombination at non-tandem direct repeats. The *ade6-L469* and *ade6-M26* or *ade6-M375* inactivating alleles were placed on either side of a functional *ura4^+^* gene. White dots represent the positions of the mutations in the *ade6* alleles. The strain is adenine auxotrophic and uracil prototrophic (ade-ura+). Different repair pathways lead to various phenotypic outcomes. Unequal sister chromatid exchange (1) produces both ade+ura- and ade-ura+ phenotypes while single strand annealing (2) or loop out (3) produces only ade+ura-. ade+ura+ phenotypes also arose for which the Whitby lab proposed a fork regression and mismatch repair model. (**B**) An assay similar to (**A**) but with the *S. cerevisiae HO* endonuclease restriction site (*MATa*) either between the *ura4^+^* and the *ade6-M26/375* alleles or in the *ade6-L469* allele. Shown are phenotypes that may arise by single strand annealing. (**C**) An assay similar to (**A**) except that *ura4^+^* was replaced with *his3^+^*. The assay was further modified by Osman et al., 2016 to introduce a nick site between the *his3^+^* and *ade6-M375* repeat. The nick is created by the M13 bacteriophage *gpII* enzyme. A replication fork which approaches from the right converts this nick into a DSB. (**D**) The assay in (**C**) was modified by introducing the *RTS1* pause site (black arrows) in between the *his3^+^* and *ade6-M375* or within the *ade6-L469* allele. Because *RTS1* is polar, two different strains were made for *RTS1*(A) and *RTS1*(B) each with a different orientation AO = active orientation, IO = inactive orientation) of the pause site. (**E**) The *RTS1* was cloned near ori1323 while the *ade-his-ade* cassette was placed at different distances (indicated in kilobases) from the *RTS1*. The relative positions of endogenous replication origins are also shown. (**F**) An assay to test collisions of replication machinery with RNA Pol III. The *tRNA^GLU^* gene was placed in either of the two orientations between the *his3^+^* and the *ade6-M375* allele while the *RTS1* pause site was placed on the right side of *ade6-M375*. (**G**) Three *LacI* arrays using the *ade-his-ade* cassette to allow microscopic visualization of repair dynamics. (**H**) An assay to study single strand annealing. Two *LEU2* fragments with overlapping regions (black arrows) were placed on either side of a functional *his3^+^* gene. The *MATa* site was placed on the 5′ end of the *his3^+^* gene. (**I**) An assay similar to (**H**) except that *his3^+^* is flanked by *ura4* with overlapping regions (arrows) instead of *LEU2*. The *MATa* site was placed on the 3′ end of *his3^+^*.

Another series of assays have been devised in *S. pombe* to study chromosomal recombination at non-tandem repeats (Figure 3). Repetitive elements had been previously shown to cause deletions, inversions or duplications [59,60]. It was known from studies in several species including *S. cerevisiae* that certain genetic mutations act as hotspots for meiotic recombination [61,62]. The *ade6-M26* (G135T) allele in *S. pombe* creates such a hotspot [63]. Remarkably, the *ade6-M375* (G132T) and the *ade6-L469* (C1467T) as well as several other alleles did not serve as recombination hotspots. The G135T, G132T and C1467T mutations inactivate the gene because they introduce stop codons.

Schuchert and Kohli designed a clever non-tandem repeat assay to study crossover frequency at the *ade6-M26* locus (Figure 3A) [64]. They positioned the *ade6-L469* 3′ end mutation on the left side and the *ade6-M26* or *ade6-M375* 5′ end mutation on the right side while placing the functional *ura4^+^* gene in between. Then, the investigators assayed for deletion or conversion. Deletion (ade+ura-) can be predicted to arise by several mechanisms. Unequal sister chromatid exchange should generate both ade-ura+ and ade+ura- phenotypes (Figure 3A(1)). A break between the two *ade6* alleles, followed by resection in both directions past the mutations and annealing can also generate a deletion through SSA (Figure 3A(2)). Since both *ade6* mutations are proximal to the *ura4^+^* gene, it is possible to reconstitute a wildtype *ade6^+^* allele. Deletion may also occur through an intra-chromosomal crossover and loopout (Figure 3A(3)). An ade+ura+ phenotype arises by replacing the mutation in one allele with the wildtype region of the other allele. The mechanism by which this happened remained elusive until later when the Whitby lab proposed a fork regression and mismatch repair model (see below).

To study mitotic recombination induced by a double strand break the Subramani group introduced the *MATa* site either in the unique sequence between *ura4^+^* and *ade6-M26/M375* or within the *ade6-L469* allele (Figure 3B) [65,66]. Upon induction of the DSB, two outcomes predominated (ade+ura- and ade-ura-) which the authors explained to arise by SSA. Subsequent genotyping by restriction digestion or backcrossing identified the exact allele in the ade-ura- phenotypes. ade+ura+ and ade-ura+ recombinants (not shown) also arose at a much lower rate and only when the *HO* restriction site was placed within the *ade6-L469* allele suggesting that the position of the break determines the repair mechanism. These assays were used to analyze the role of several recombination genes in DSB repair [65].

The Whitby lab has subsequently been influential in designing several variations of the tandem repeat assay and have been able to explain some of the more complex outcomes. These assays have been used to identify and characterize genes involved in DNA damage repair [67,68,69,70,71,72,73,74,75,76,77,78]. The investigators initially designed a system similar to the Schuchert and Kholi assay except that instead of *ura4^+^* they placed a *his3^+^* marker between the *ade6* alleles (Figure 3C) [79]. This assay was used to show that the ura+his+ recombination outcomes may arise due to recombination dependent restart of stalled replication forks usually through BIR. Inactivation of various cellular processes such as Holliday junction resolution or the checkpoint changes the recombination outcomes [70,79,80,81,82]. In a more recent report [83], the Whitby lab introduced a nicking site for the *gpII* M13 phage enzyme. This enzyme makes a nick that is converted to a DSB by the replication fork which approaches from the right (see Figure 3D,E) [84,85]. This assay was used to study restart of stalled replication forks due to the DSB produced by this nick.

To further investigate the effect of replication fork stalling on recombination outcomes, the Whitby lab modified the assay by placing *RTS1*, the naturally occurring replication fork termination site at the mating type loci [86], either between the *his3^+^* and the *ade6-M375* alleles or within the *ade6-L469* allele (Figure 3D) [87,88]. *RTS1* is polar meaning that it can only stall forks in one direction, so strains were constructed with different *RTS1* orientations, *RTS1-AO* (active orientation) and *RTS1-IO* (inactive orientation). A cluster of origins of replication are found to the right of the *ade6-M375* allele and the replication fork is predicted to approach from the right (Figure 3E). Two-dimensional gel electrophoresis showed that the *RTS1-AO* efficiently stalls replication forks [89]. The investigators used this assay to study the function of various recombination genes in fork restart and to show that rescue of collapsed replication forks can cause BIR dependent template switching that can generate chromosomal rearrangements. [76,87,88,90,91]. A protocol was published with extensive details on the use of these elegant assays [92].

By inserting the *ade-his-ade* cassette at different distances from the *RTS1* pause site (Figure 3E) the investigators showed that template switching can occur up to 75 Kb from collapsed forks [93]. Collisions between Pol III which transcribes tRNA and replication can cause chromosomal rearrangements [94]. A *tRNA^GLU^* gene was inserted between the *his3^+^* and the *ade6-M375* allele to show that collisions between Pol III and replication machinery increases the frequency of recombination (Figure 3F) [91,93]. An increase in template switching was observed when both *RTS1* and *tRNA^GLU^* were introduced in the same construct and the orientation of transcription of *tRNA^GLU^* faced *RTS1* head on [93]. To monitor interaction of fluorescently tagged recombination genes (*rad52^+^*, *rad51^+^*, etc.), the *LacO* array was also introduced at different positions within the *ade-his-ade* repeat (Figure 3G) [88,89,95,96].

An assay to study SSA in *S. pombe* was designed by Watson et al. (Figure 3H) [97]. Two *S. cerevisiae LEU2* fragments with overlapping regions were placed on either side of a functional *his3^+^* gene. The MATa sequence was cloned at the 5′ end of *his3^+^* right before the start codon. Using this assay, the investigators showed that SSA is *rad52^+^* dependent, confirming previous findings. In their report Watson and colleagues also designed an elegant system that allows fast transcriptional induction in *S. pombe*. Historically, transcriptional induction in *S. pombe* relied on the *nmt1* promoter which is repressed by thiamine. Removal of thiamine de-represses the promoter but it takes anywhere between 14–20 h for full induction [98,99]. The new system which is based on upregulation of the *urg1* promoter allows induction within 30 min mirroring the *S. cerevisiae GAL* induction system [100]. The *urg1* system was optimized in a subsequent publication [101]. Other systems for faster induction of gene expression in *S. pombe* that we do not discuss here have also been engineered more recently [102,103].

We also designed an assay to study intrachromosomal deletions at direct repeats (Figure 3I) [31,104]. A functional *his3^+^* gene was placed between two truncated *ura4* alleles with 200 bp of overlapping sequence. We showed that this assay can only detect deletions and not conversion.

## 4. Chromosomal Rearrangements Caused by Stalled or Collapsed Replication Forks at Inverted Repeats

**Figure 4:**
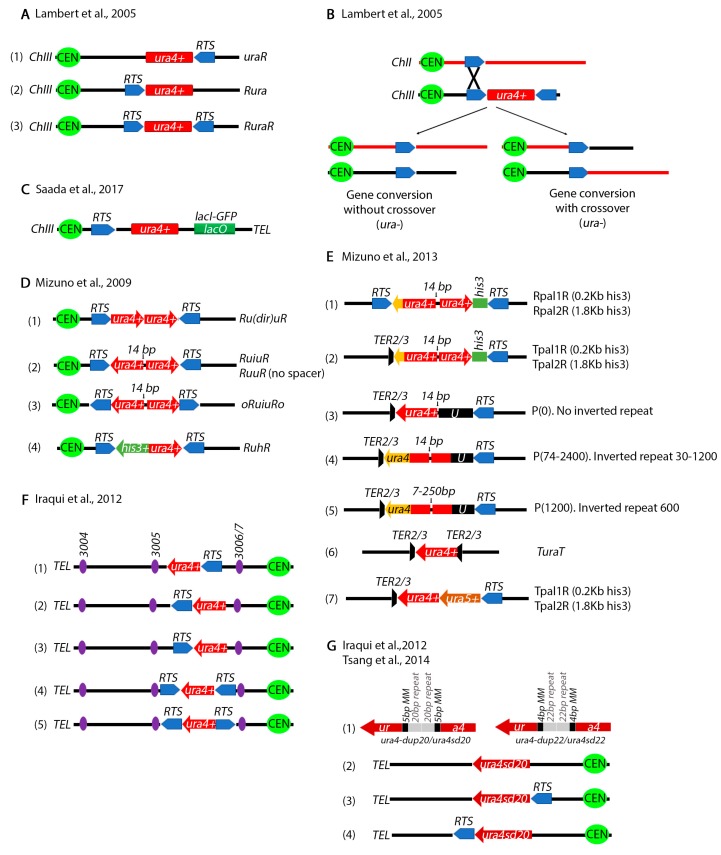
Assays to study gross chromosomal rearrangements at inverted repeats. (**A**) An assay to study fork stalling at repetitive elements. (1) The *RTS1* pause site was placed on the right of a functional *ura4+* gene (*uraR*), (2) left (*Rura*) or (3) both sides (*RuraR*). Fork stalling was assayed using pulse field gel electrophoresis (PFGE). (**B**) Chromosomal rearrangements of constructs in (**A**) can occur by two mechanisms: gene conversion without crossover and gene conversion with crossover. In both cases there is a recombination event between the *RTS1* site next to the *ura4^+^* cassette and the endogenous *RTS1* at the *MAT* locus on Ch.II. Both types of events result in loss of *ura4^+^*. (**C**) Modification of construct in (**A**) to monitor chromosomal dynamics by fluorescence microscopy. A *LacO* array was placed on the telomere side of the *RTS1/ura4^+^* cassettes. (**D**) Repetitive elements constructs to study chromosomal rearrangements. (1) *RTS1* cassettes were placed on both sides of two directional *ura4^+^* repeats (*Ru(dir)uR*). (2) *RTS1* was placed on both sides of inverted *ura4^+^* repeats spaced by 14 bp of unique sequence (*RuiuR*). A construct without the 14bp spacer was also made (*RuuR*) (3) In *oRuiuR* the *RTS1* is in opposite direction from the *RuiuR* construct. (4) In the *RuhR* construct, one of the *ura4^+^* repeats was replaced with *his3^+^*. (**E**) Several constructs to test the effect of repeat length and the position of the replication pause sites in producing rearrangements. (1) Two *ura4^+^* inverted repeats spaced by 14 bp of unique sequence were engineered with 0.2 Kb (*Rpal1R*) or 1.8 Kb (*Rpal2R*) *his3^+^* sequence between the right *ura4^+^* repeat and the *RTS1* pause site. An additional short *ura4^+^* was placed between the left *RTS1* and the *ura4^+^* repeat (yellow arrow). (2) *Tpal1R* and *Tpal2R* constructs are similar to constructs in (1) except that the left *RTS1* was replaced with three *TER2/3* sequences. (3) In this construct the right *ura4^+^* repeat was replaced by a unique sequence. (4) Each of the *ura4^+^* repeat sizes were varied between 30–1200 base pairs. (5) The spacer between the inverted repeats was varied between 7–250 base pairs. (6) *ura4^+^* cassette flanked by the *TER2/3* termination sites. (7). Direct repeats but one of the *ura4^+^* repeats was replaced with a *ura5^+^* repeat. (**F**) The *ura4^+^* and *RTS1* pause sites were placed next to known origins of replication (3004, 3005, 3006/7). (1) Co-directional *ura4^+^* and *RTS1* with *RTS1* placed either after (1) or in front (2) of the *ura4^+^* repeat. (3) Construct similar to (**A**) (2) and (4) construct similar to (**A**) (3) placed between the 3005 and 3006/7 replication origins. (5) Construct similar to (4) but with *RTS1* in the opposite orientation. (**G**) Constructs to test microhomology mediate repair caused by rescued replication forks. (1) Two different microhomology constructs. *ura4-dup20*/*ura4sd20* was engineered by placing two 20 bp (grey boxes) repeats flanked by 5 bp of microhomology sequences (black boxes) in the middle of the *ura4^+^* gene. *ura4-dup22*/*ura4sd22* is has 22 bp repeats flanked by 4 bp of homology. Microhomology mediated repair was investigated either in the absence of a pause site (2) or with the pause site placed on the left (3) or right (4) of the construct. Only *ura4sd20* is shown.

A series of other assays have been designed to study chromosomal rearrangements resulting from stalled replication forks by the Carr, Murray and Lambert labs. Carr and colleagues placed the *RTS1* on either side of *ura4^+^* gene on Ch.III (Figure 4A) [105]. Using 2-D gel electrophoresis they showed that these constructs can efficiently stall forks in the vicinity of the *ura4^+^* gene. Deletion of several recombination genes including *rad51^+^* decreases cell viability suggesting that homologous recombination is required for rescue of stalled forks. PFGE and PCR showed that some of the outcomes resulted in *ura4^+^* loss through gene conversion without crossover, while others through a crossover between Ch.III and Ch.II produce a reciprocal translocation (Figure 4B). In all cases, information was exchanged between Ch.III *RTS1* and the endogenous Ch.II *RTS1*. To monitor anaphase bridges by microscopy, the Lambert lab also placed a *LacO* array next to the *RTS1* pause site (Figure 4C) [106].

Mizuno et al. engineered several repeat constructs flanked by *RTS1* sites (Figure 4D) [107]. Using these constructs, the investigators showed that recombination dependent rescue of stalled replication forks at inverted repeats can produce dicentric and acentric isochromosomes. In subsequent and even more sophisticated studies, the investigators generated a series of more complex constructs to precisely analyze the mechanisms of chromosomal rearrangements at inverted repeats (Figure 4E) [108]. The length of the repeats as well as the gap between the repeats was varied, unique sequences were introduced at either side of the repeats, *RTS1* sites were placed at different distances from the repeats and the *TER2/3* ribosomal fork barrier was also tested. Their findings showed that inverted repeats cause forks to turn around or “execute a U-turn” and generate gross chromosomal rearrangements. Remarkably, the DNA damage checkpoint does not appear to detect the recombination intermediates that cause these rearrangements, at least in the cell cycle in which they occur [109].

The position of the *RTS1* was varied relative to several Ch.III replication origions (Figure 4F) [110,111] to show that when a replication fork approaching from one of these origins collides with the *RTS1* site, it causes deletions in addition to gross chromosomal translocations. To test for replication fork slippage, the investigators designed a construct where the *ura4^+^* gene was interrupted by 20 bp repeats flanked by 5 bp of microhomology sequences (Figure 4G) [77,110]. A slightly modified construct has 22 bp repeats flanked by 4 bp of microhomology (Figure 4G). Both constructs inactivate the *ura4^+^* gene. Microhomology mediated repair results in a functional *ura4^+^*. Using these constructs, it was shown that fork slippage, but not translesion synthesis or mismatch repair, is responsible for the restoration of the functional *ura4^+^* cassette. The intra-S phase DNA damage checkpoint (Rad3) represses fork slippage and microhomology mediated repair at stalled forks [77]. Further, the NHEJ factor Ku appears to regulate recombination at arrested forks by controlling end resection [112].

## 5. Mating Type Loci Serve as a Natural Site for Studying Collapsed Replication Forks

**Figure 5:**
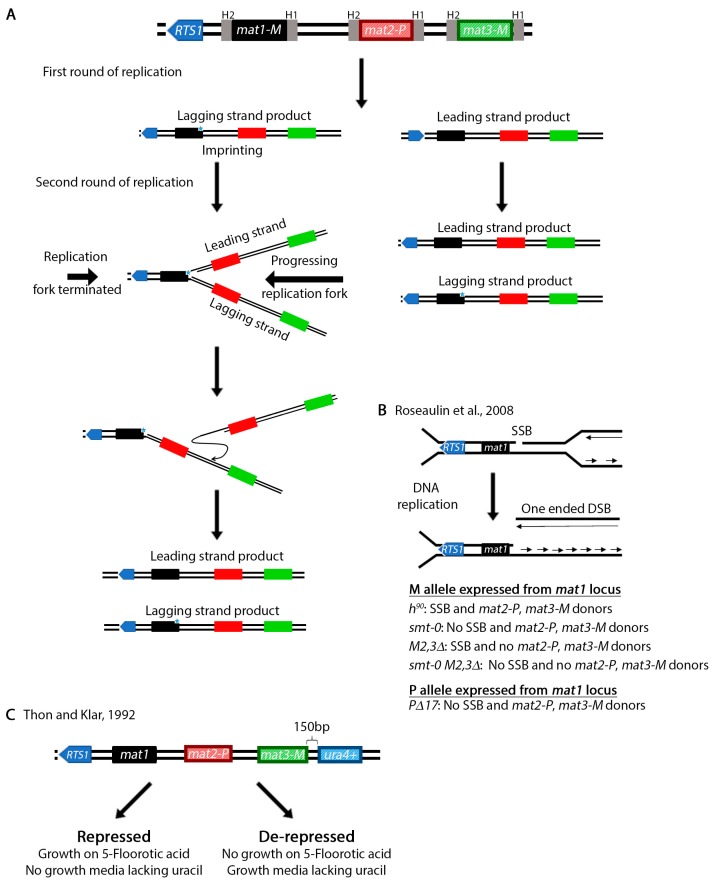
Mating type switching in *S. pombe*. (**A**) Diagram of the process of mating type switching. The mating type region has three cassettes *mat1*, *mat2-P(plus)* and *mat3-M(minus).* Only *mat1* is expressed with either P or M while *mat2-P* and *mat3-M* are silenced. The shading of *mat2-P* and *mat3-M* diagrams represents this silencing. Information switches from *mat2-P* and *mat3-M* onto *mat1* so that *mat1* expresses either P or M. All three cassettes are flanked by homologous regions (H1, H2) that facilitate recombination. In this diagram, we begin with a hypothetical example where *mat1* expresses the *M* information. After one round of replication, an imprint consisting of insertion of one or two ribonucleotides or a single strand nick is made next to the *mat1-M* cassette on the lagging strand (blue star). For simplicity, the top diagram does not have the imprint. In the second round of replication, the leading strand collides with the imprint and produces a blunt one-ended DSB. To ensure that only the leading strand passes through the imprint, the replication fork from the other direction is paused and terminated by the *RTS1* site. The one-ended break is repaired by copying information from the *mat2-P* or *mat3-M* regions. Since in our example the *M* information was expressed at *mat1*, the break may be repaired using *mat2-P* and the information at *mat1* switches. Note that the imprint is always placed on the lagging strand with each DNA replication. (**B**) Exploiting the mating type loci to study rescue of collapsed forks. Collision of the leading strand with the imprint produces a blunt one-ended break that can be studied in WT and other control strains. *h^90^* is a wild type strain capable of switching that represents a mix of M and P cells. The *smt-0* has a deletion that eliminates the imprint while *M2,3Δ* have deletions in the *mat2-M* and *mat3-P* regions. *smt-0 M2,3Δ* is a double mutant. The *PΔ17* mutant is similar to *smt-0* except that in this strain the *mat1* locus expresses the *P* information. (**C**) An assay to study silencing at the mating type loci. The functional *ura4^+^* gene was places 150 base pairs from *mat3-M*. If repressed, the strain can grow on 5-fluoroorotic acid (5-FOA) which negatively selects against uracil prototrophs and die on minimal media lacking uracil. If de-repressed, the strain will die on 5-FOA and grow on minimal media lacking uracil.

The mating type of a *S. pombe* cell is determined by the allele present at the *mat1* cassette (Figure 5A) [113]. This allele can be either *mat1M* (M cell, M stands for minus) or *mat1P* (P cell, P stands for plus) [114,115]. Switching between *mat1M* and *mat1P* is accomplished by copying information from the silent *mat2-P* and *mat3-M* cassettes [116,117,118,119]. In addition to the allele present at the *mat1* locus, the mating type of a population of cells is also determined by the ability to switch and the alleles present at the silent *mat2-P* and *mat3-M* regions. Wild type h^90^ cells can switch information at the *mat1* locus and are mixture of M and P cells [120,121,122,123]. A population that has the M allele at the *mat1* locus (*mat1M*) and lost the ability to switch is h^-^. A population may also be h^-^ if it lost the *mat2-P* locus and repairs the *mat1M* with the same information (e.g., from *mat3-M*). Alternatively, a population of cells are h^+^ if the P allele is expressed from the *mat1* locus (*mat1P*) and the strain is unable to switch (e.g., *mat1PΔ17*).

In wild type populations, switching occurs in only 25% of the cells [124]. This is because unlike in *S. cerevisiae* where switching is initiated by a double strand break generated by the *HO* endonuclease [125], in *S. pombe* switching begins with an imprint in the lagging strand during an initial round of DNA replication. Two concurrent models exist to explain the nature of the imprint. The imprint may be caused by the introduction of one or two ribonuclotides in the DNA sequence [126,127,128] or the generation of a single strand break [129] or possibly both at the same time. Regardless, the imprint is converted to a one-ended double strand break in the next round of replication [129,130,131,132,133]. However, only one of the replication forks may convert the imprint into a DSB. To ensure that the other replication fork does not pass through the imprint, replication is terminated by the above mentioned *RTS1* site [86,134]. This process allows unidirectional transfer of information from the two *mat2-P* and *mat3-M* cassettes onto *mat1* in only one of the four cells.

Investigation of the mating type loci in *S. pombe*, which increased our understanding of replication, recombination and gene silencing has been reviewed previously [120,135]. This natural system is a remarkable assay for the study of the role of DNA recombination in restart of collapsed replication forks. Perhaps even more important is the fact that the system produces one-ended DSBs which more accurately resemble the type of substrates resulting from collapsed replication forks [136]. Using wild type cells or several mating type defective mutants (Figure 5B) [130,137,138] the role of various DNA damage response genes have been investigated [136,139,140]. Work has also led to the identification of several recombination and replication genes, particularly the *swi* (switching) genes [117,118,141,142]. Additionally, various mechanisms of gene silencing were identified (reviewed in reference [143]). For example, unlike the centromeres, the mating type loci are silenced through an RNAi independent mechanism [144,145,146]. Work on silencing led to the identification of the *clr* (cryptic loci regulator) genes [147,148,149]. To investigate silencing, Thon and Klar placed the *ura4^+^* gene 150 bp from the *mat3* cassette [147] (Figure 5C). Finally, as Klar et al. point out in their review [135], the asymmetric mating type switching in *S. pombe* may also explain how genes are differentially regulated during development of higher eukaryotic organisms.

## 6. Other Fluorescence and Biochemical Assays

**Figure 6:**
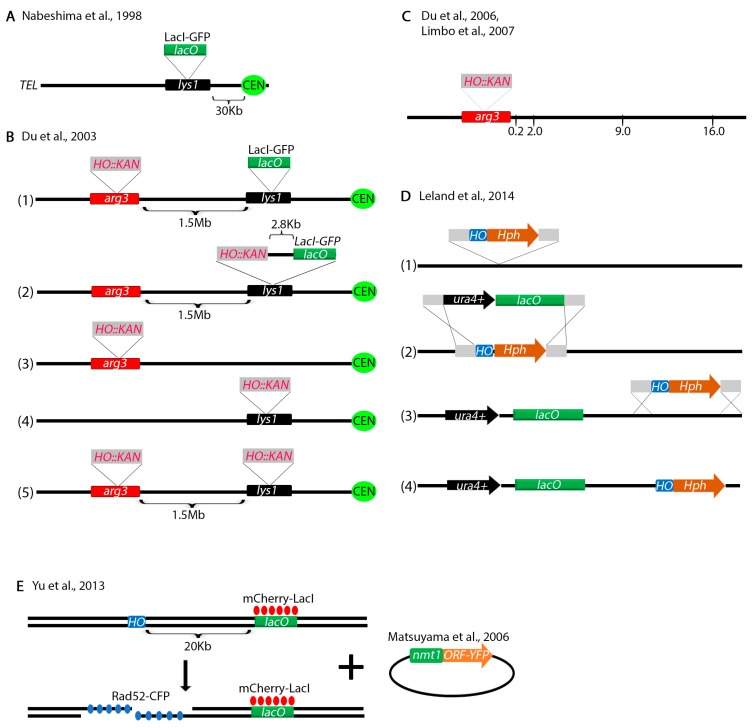
Fluorescence and biochemical assays. (**A**) An assay designed by Nabeshima et al. to study centromere dynamics. A *LacO* array was placed 30 Kb from the centromere of Ch.I. *LacI-GFP* binding allows visualization of centromeres. (**B**) Assays developed by Du et al. to study repair of a DSB. (1) The *HO* endonuclease restriction site marked with *KAN* was placed 1.5 Mb away from the *LacO* array at the *arg3^+^* locus. (2) *HO::KAN* was placed 2.8 Kb from the *LacO* array. Constructs without a *LacO* array with the *HO* restriction site at *arg3^+^* (3), *lys1^+^* (4) or both *arg3^+^* and *lys1^+^* (5) were also engineered. These constructs can be used to study foci of fluorescently tagged DNA damage repair proteins. (**C**) The construct in (**B**) (3) was adapted for Chromatin Immunoprecipitation. PCR primers were engineered at 0.2, 2.0, 9.0 and 16.0 Kb from the break. Upon induction of the break, multiplex PCR can analyze binding of various proteins at all four locations at once. (**D**) An assay to integrate the *LacO* array and the *HO* restriction site anywhere in the yeast genome. Please see text for details. (**E**) An assay to study YFP tagged proteins interacting with a DSB. A *lacO* array that interacts with LacI-mCherry was placed 30 Kb from the *HO* endonuclease restriction site. The strain also contains Rad52 fused with cyan fluorescent protein (Rad52-CFP). A library of YFP tagged proteins was transformed in the cells and colocalization of all three foci (red, blue, yellow) was monitored.

To investigate centromere dynamics, Nabeshima et al. placed a *LacO* array at the *lys1^+^* locus, 30 Kb from the centromere (Figure 6A) [150]. The Russell lab modified this assay by placing the *HO* endonuclease restriction site 1.5 Mb away at the *arg3^+^* locus within 2.8 Kb of the *LacO* array (Figure 6B) [151]. Using these assays, they showed that Crb2-YFP (yellow fluorescent protein) co-localizes with the *LacI-GFP* (green fluorescent protein) bound to the *LacO* array suggesting that Crb2 interacts with the chromosome at the site of the DSB. When the break was at the *arg3^+^* locus, no co-localization was observed because the Crb2-YFP interacts with a DNA sequence too far from the *LacO* array. Several other constructs were made without the *LacO* array to investigate foci of various fluorescent-tagged proteins in response to a DSB (Figure 6B). For finer analysis of repair proteins interacting with the break, the investigators next turned to Chromatin IP. Primers were designed to amplify sequences by PCR at 0.2, 2.0, 9.0 and 16.0 Kb and the interaction of a variety of proteins with the break were tested biochemically (Figure 6C) [152,153]. Using these assays, the investigators have unraveled the role of various DNA damage checkpoint and repair proteins in processing of DSBs [136,152,154,155,156,157,158,159,160]. Not discussed here are some other biochemical assays that the Russell lab engineered, including some assays to study resection [161] with some adaptations from *S. cerevisiae* [162].

Another ingenious assay was designed by Leland and King to introduce a *LacO* array and the *HO* restriction site anywhere in the genome (Figure 6D) [163]. This technique requires several steps. First, the *HO* restriction site marked with the *HPH* marker and flanked by two homology regions is placed at a desired location in the genome (Figure 6D(1)). Then, a sequence harboring the *LacO* array marked with *ura4^+^* and flanked by the same homologous sequences as the *HO* restriction site is integrated at the *HO* locus (Figure 6D(2)). The *HO* restriction site is re-integrated at a location neighboring the *LacO* array (Figure 6D(3)). This produces a construct with an *HO* restriction site next to the *LacO* array (Figure 6C(4)) similar to the Du et al. assay (Figure 6B). The advantage of this technique is that the *LacO* and the *HO* restriction site can be introduced anywhere in the genome.

Yu et al. has designed an assay to screen for proteins interacting with a DSB (Figure 6E) [164]. They engineered the *HO* endonuclease restriction site 20 Kb from a *LacO* array that binds LacI-Cherry. The strain also expresses Rad52-CFP (cyan fluorescent protein) which interacts with DSBs. Upon DSB induction, co-localization of red and blue foci indicates that Rad52-CFP interacted with the DSB. When they transformed a library of *S. pombe* YFP tagged proteins [165], they were able to screen for other proteins that interact with the DSB by monitoring co-localization of all three foci (red, blue, yellow).

## 7. Non-Homologous Repair

**Figure 7:**
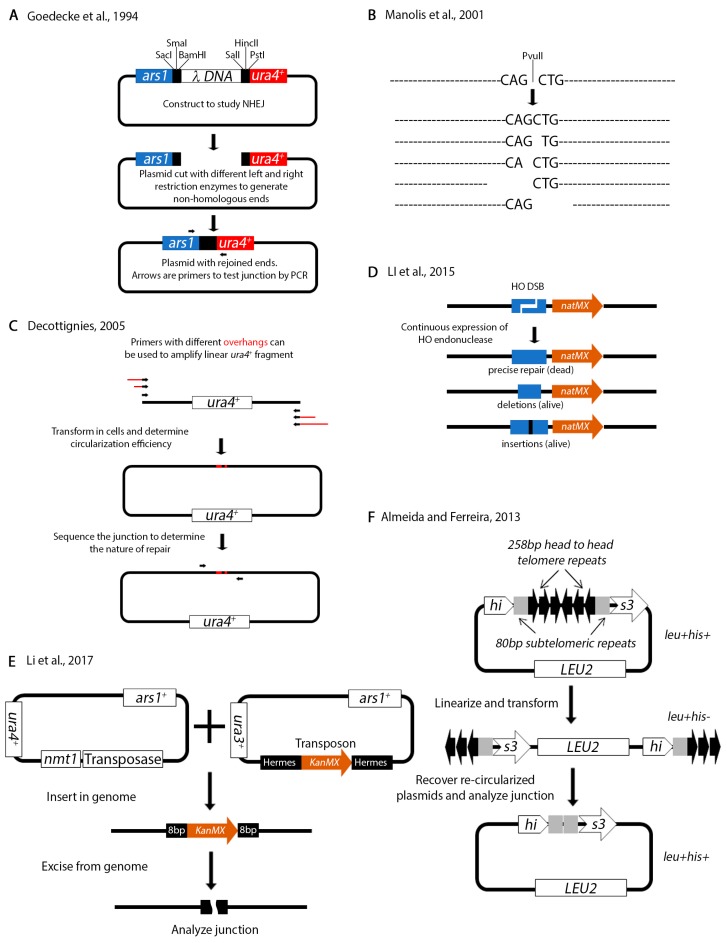
Assays to test non-homologous repair. (**A**) A plasmid-based assay to study non-homologous end joining. A λ DNA fragment is flanked by three unique restriction sites on each side. The plasmid is cut with different left and right restriction enzymes then transformed as a linear fragment into cells. Propagation of the transformed fragment in cells is only possible upon re-ligation of linear fragment ends. Thus, transformation frequency can be used as an indicator of repair efficiency. PCR across the junction (black arrows represent primers) followed by sequencing can determine the ligation patterns. (**B**) An assay similar to (**A**) except that the plasmid is cut with only one enzyme (*Pvu*II). After relegation, the junction is analyzed for reconstitution of the *Pvu*II restriction site or deletion and insertion of various nucleotides. (**C**) In this assay, PCR primers (black arrows) can be designed with various sequence overhangs (red lines). Linear fragments are amplified and transformed in yeast. Repair is analyzed as in (**A**). (**D**) The *HO* restriction site marked with *nat*MX was cloned at the *arg1^+^* locus on Ch.I. Continuous expression of *HO* endonuclease will kill all cells that do not repair the break or repair it correctly because it reconstitutes the restriction site and are vulnerable to re-cutting. Incorrect repair destroys the restriction site and allows cells to live. The authors used both Sanger sequencing and Illumina next generation sequencing to analyze the junction. (**E**) A transposon-based assay to study genome integration and excision. A plasmid encoding the transposase is co-transformed with another plasmid with transposon integration sequences (Hermes) flanking a KanMX cassette. De-repression of the transposase allows integration of the KanMX cassette randomly in the genome. Following integration, re-expression of the transposase will cause excision of the transposon. Analysis of the junction sequences can determine the form of repair. (**F**) An assay to test the role of telomeres in preventing chromosome end fusions. Two head to head telomeric repeats were cloned in the second intron of the *his3^+^* gene. Each repeat also contains 80 bp of subtelomeric sequences. This telomere repeat interruption does not disrupt transcription of the *his3^+^* gene. Consequently, the plasmid is his+leu+. The plasmid is linearized (destroying transcription of *his3^+^*) between the telomeric repeats and introduced into cells. Because each end is capped by telomeres, the fragment can be maintained as a mini-chromosome, which is leu+his-. Deletion of telomerase causes telomere attrition and fusion of the ends which reconstitutes the *his3^+^* gene. Such fusions can be selected for on media lacking both histidine and leucine. Analysis of the fusion junction can determine the precision of repair.

Non-homologous repair can occur in the absence of homologous regions. Several assays have been designed to test non-homologous repair in *S. pombe*. In an NHEJ assay designed by Goedecke and colleagues, a plasmid is linearized with various restriction sites to produce non-homologous ends (Figure 7A) [166]. The restriction sites used to generate one of the ends are different from those used to generate the other end, thus ensuring that the ends cannot be re-ligated. The linear fragment is transformed into living cells and allowed to re-circularize. The junction is amplified by PCR and sequenced. Using this assay, the investigators showed that the ends are resected up to 14 bps prior to rejoining. A similar assay by Manolis et al. relies on transforming linearized sequences with just one restriction enzyme (*Pvu*II) (Figure 7B) [167]. Subsequent sequence analysis of rejoined products identified various sequence alterations at the junction. The investigators also showed that in *S. pombe rad50^+^*, *mre11^+^* and the DNA damage checkpoint is not required for NHEJ. These plasmid type NHEJ assays were instrumental in demonstrating that key components of NHEJ are conserved form yeast to humans [168,169].

In a variation of these assays, Anabelle Decottignies used PCR to amplify a linear fragment (Figure 7C) [170]. The fragment was transformed and allowed to re-circularize in living cells followed by junction analysis. Remarkably, she identified mitochondrial DNA sequences at the junctions. As she points out, the advantage of this assay is that primers with different microhomology overhangs can be engineered to test recircularization of different ends. This assay was successfully used by the Du lab to identify the XRCC4 NHEJ protein in a genome wide screen [171]. The Du lab also designed an assay that relies on the *HO* restriction enzyme (Figure 7D) [172] similarly to their previous assays (Figure 6B). In this assay, the 24 base pair *HO* restriction site marked with the *natMX* cassette (*nat* confers resistance to nourseothricin) was cloned at the *arg3^+^* locus on Ch.I. The *nmt1-HO* endonuclease was integrated at *ars1^+^*. Because the cells were grown under continuous expression of the *HO* endonuclease (no thiamine in the media), only those cells with imprecise repair of the junction (either deletions or insertions) could survive. These cells had destroyed the *HO* endonuclease restriction site, in effect inactivating the function of the enzyme. The junction of surviving cells was amplified by PCR and sequenced either by the Sanger methods or by Illumina sequencing. This assay is a useful tool for studying break repair that does not rely on homologous recombination.

A different NHEJ assay was designed by Li et al. that relies on analyzing the genome insertion and excision of a transposon (Figure 7E) [173]. Two plasmids, one encoding the transposase and the other the transposon are co-transformed in the cells. De-repression of the transposase causes random insertion of the transposon. The role of NHEJ factors can be investigated by monitoring the efficiency of insertion. Alternatively, re-expression of the transposase will cause excision of the transposon from the genome. The transposon is characterized by 8 bp of homology on either end. Analysis of the repaired junction can determine the nature of the microhomology mediated repair.

Finally, we want to mention an assay to study chromosome end fusions due to telomere erosion (Figure 7F) [174]. The *his3^+^* is one of the genes in *S. pombe* that has introns. The investigators introduced head to head telomeric repeats in the second intron of *his3^+^*. This insertion does not affect the function of *his3^+^*. The plasmid is linearized between the telomeric repeats and transformed into living cells. The construct may be propagated as a linear fragment because it has telomeres and can be selected for on media lacking leucine. However, the linear fragment interrupts transcription of the *his3^+^* gene. Deletion of telomerase causes telomere attrition and re-circularization of the fragment which can be selected for on media lacking histidine because it reconstitutes the function of the *his3^+^* gene. The plasmid can be recovered and the junction analyzed by sequencing.

## 8. Concluding Remarks

Although none of the assays mentioned here can independently unravel the function of every repair pathway, when the data are put together, a clearer picture emerges. This is particularly true if combined with results from other systems such as *S. cerevisiae*. Nevertheless, the work done in *S. pombe* has not just been complementary to the work done in other model systems but instrumental in discovering and defining new mechanisms of DSB repair.

Nevertheless, there are many unanswered questions that remain. For example, the role of chromatin remodeling in modulating DSB repair requires further investigation. The fact that histone modifications play a role in biasing repair towards different pathways is clear. However, how these modifications choreograph repair is not well understood [175]. This is because histone modifications are transient and hard to capture. Experiments using some of the assays described here that either knock out histone modifying enzymes or substitute modifiable histone residues for unmodifiable ones have provided some insight but these are likely to cause genome wide changes which complicates interpretation. Some recent experiments have also shown that RNA plays a major role in DSB repair [176] but the exact dynamics are poorly understood. With advances in technology, such as CRISPR, it is almost certain that in the next few years we will see much more complex assays that are likely to elucidate some of these questions.
